# Comparative Genomic Screen in Two Yeasts Reveals Conserved Pathways in the Response Network to Phenol Stress

**DOI:** 10.1534/g3.118.201000

**Published:** 2019-01-15

**Authors:** Bashar Alhoch, Alan Chen, Elaine Chan, Asmaa Elkabti, Sasha Fariña, Catherine Gilbert, Jean Kang, Bradley King, Karen Leung, Julia Levy, Elizabeth Martin, Benjamin Mazer, Sara McKinney, Alexandra Moyzis, Margaret Nurimba, Michelle Ozaki, Kathleen Purvis-Roberts, Joshua Marc Rothman, Shravya Raju, Cynthia Selassie, Oliver Smith, Julia Ticus, Gretchen Edwalds-Gilbert, M. Cristina Negritto, Ruye Wang, Zhaohua Tang

**Affiliations:** * W.M. Keck Science Department, The Claremont Colleges, Claremont, CA 91711; † Molecular Biology Program, Pomona College, Claremont, CA 91711; ‡ Chemistry Department, Pomona College, Claremont, CA 91711; § Engineering Department, Harvey Mudd College, Claremont, CA 91711

**Keywords:** comparative genomic screen, phenol stress response in yeasts, cell cycle regulation and DNA repair, ergosterol and V-ATPase, UPR

## Abstract

Living organisms encounter various perturbations, and response mechanisms to such perturbations are vital for species survival. Defective stress responses are implicated in many human diseases including cancer and neurodegenerative disorders. Phenol derivatives, naturally occurring and synthetic, display beneficial as well as detrimental effects. The phenol derivatives in this study, butylated hydroxyanisole (BHA), butylated hydroxytoluene (BHT), and bisphenol A (BPA), are widely used as food preservatives and industrial chemicals. Conflicting results have been reported regarding their biological activity and correlation with disease development; understanding the molecular basis of phenol action is a key step for addressing issues relevant to human health. This work presents the first comparative genomic analysis of the genetic networks for phenol stress response in an evolutionary context of two divergent yeasts, *Schizosaccharomyces pombe* and *Saccharomyces cerevisiae*. Genomic screening of deletion strain libraries of the two yeasts identified genes required for cellular response to phenol stress, which are enriched in human orthologs. Functional analysis of these genes uncovered the major signaling pathways involved. The results provide a global view of the biological events constituting the defense process, including cell cycle arrest, DNA repair, phenol detoxification by V-ATPases, reactive oxygen species alleviation, and endoplasmic reticulum stress relief through ergosterol and the unfolded protein response, revealing novel roles for these cellular pathways.

Eukaryotic cells have evolved elaborate mechanisms to precisely sense and efficiently respond to various environmental perturbations arising from internal and external insults. Cells respond to stress in a variety of ways ranging from activating repair pathways for cell recovery to eliciting programmed cell death for elimination of damaged cells. A pathological stress response is a hallmark of many common human diseases including cancer, diabetes, and inflammatory disorders, as well as several neurodegenerative disorders, such as Alzheimer’s disease, Parkinson’s disease, and amyotrophic lateral sclerosis (ALS) (Fulda *et al.* 2010; Karam *et al.* 2015). Signaling pathways have been identified that are activated in response to various stress conditions including alterations in nutrients, temperature, pH, osmolarity, and reactive oxygen species (ROS); however, what the connections among pathways are and how they integrate into a coordinated network for stress response, remain poorly understood as fundamental questions in stress biology (Ho and Gasch 2015).

Phenols comprise a diverse class of naturally abundant and synthetic compounds that are widely employed by the pharmaceutical and chemical industries. Butylated hydroxyanisole (BHA), butylated hydroxytoluene (BHT), and bisphenol-A (BPA) are three phenol model molecules used in our study. BHA, BHT, and BPA share a phenol unit but have either distinct functional groups or specific arrangements of some common groups with the benzene ring. BHA and BHT are used as food additives due to their antioxidant properties (Williams *et al.* 1999), while BPA is a high production-volume chemical for manufacture of polycarbonate plastic and epoxy resins, and present in many household items (Hormann *et al.* 2014). Conflicting reports regarding their anti-oxidant and genotoxic properties exist. A recent study using *S. cerevisiae* reported that BHA, BHT, and BPA are cytotoxic and BHA is a potential carcinogen (Negritto *et al.* 2017); however, little has been explored regarding global signaling pathways responsible for biological consequences of the phenol derivatives.

In this study, we took a comparative genomics approach with two divergent yeasts to investigate the conserved response pathways to phenol derivatives as stress molecules, exploring the molecular connections among these pathways. We have identified a large number of genes required for coping with the phenol stress for cell survival. The results provide a global picture of the cellular defense process, including stress-activated protein kinase signaling, cell cycle block, DNA damage and repair, phenol detoxification by V-ATPases, intracellular ROS reduction through ergosterol synthesis, and cellular stress alleviation by the unfolded protein response (UPR) in the endoplasmic reticulum (ER), representing novel functions of these known cellular pathways. Significantly, the genes identified in this comparative genomics approach are enriched in disease-associated human orthologs, which underscores the important cellular processes to consider when evaluating the human health consequences of phenol derivatives.

## Materials And Methods

### Strains and deletion libraries

Routine yeast cell culture and standard genetic methods were carried out as described previously (Moreno *et al.* 1991; Alfa *et al.* 1993; Burke *et al.* 2000; Forsburg and Rhind 2006; Tang and Edwalds-Gilbert 2016). *S. pombe* and *S. cerevisiae* strains were grown in rich medium, yeast extract plus supplements (YES) or yeast extract peptone dextrose (YPD), respectively, or minimal medium (MM) with appropriate supplements. Cells were incubated at 30° unless otherwise specified. The *S. pombe* haploid deletion mutant library was purchased (Bioneer, Ver. 1.0). The Instruction Manual of Bioneer was used as a guideline for the growth and maintenance of these deletion strains. Other *S. pombe* strains used are listed in Table 1. The *MAT***a** *S. cerevisiae* deletion library and corresponding wild-type (*wt*) strain were purchased from Open Biosystems. The library was replicated and stored according to Open Biosystems directions. Other *S. cerevisiae* strains used are listed in Table 2.

**Table 1 S.** *pombe* Strains Used

Strain	Genotype	Source or reference
972	*h^-^*	Yanagida, Kyoto University
1913	*h ^-^ leu1-32*	Dunphy, Caltec
501	*h ^-^ ade6-704 leu1-32ura4-D18*	Carr, University of Sussex
JMM1179	*h^-^ ade6-704 leu1-32 ura4-D18 rad22-GFP*::*kanMX6*	Carr, University of Sussex

**Table 2 S.** *cerevisiae* Strains Used

Strain	Genotype	Source
W3749-14C	*MAT***a**, *ADE2*, *bar1*::*LEU2*, *trp1-1*, *LYS2*, *RAD52-YFP*, *RAD5*	Rothstein Lab*
ABX367-3A	*MAT***a**, *ade2-1*, *can1-100*, *leu2-3*, *112*, *trp1-1*, *ura3-1*, *HIS3*	Negritto Lab**
TNT37-20	*MAT*α, *ade2-1*, *can1-100*, *leu2-3*, *112*, *trp1*::*HIS3*::*GAL*::*HO*, *ura3-1*, *his3-1 or his3-∆BglII*::*HOcs?*, *sir3*::*TRP1*	Negritto lab
TNT45-3	*MAT*α, *ade2-1*, *can1-100*, *leu2-3*, *112*, *trp1*::*HIS3*::*GAL*::*HO*, *ura3-1*, *his3-1 or his3-∆BglII*::*HOcs*, *sir3*::*TRP1*, *sir4*::*KNMX*	Negritto lab
TNT48-6	*MAT*α, *ade2-1*, *can1-100*, *leu2-3*, *112*, *trp1-1*, *ura3-1*, *HIS3*, *sir2*::*TRP1*, *sir3*::*KNMX*	Negritto lab
ABX1796-57D	*MAT*::*LEU2*, *ade2-1*, *can1-100*, *leu2-3*, *112*, *trp1*::*GAL*::*HO*::*KNMX*, *ura3-1*, *RAD52-FLAG-KNMX*, *dnl4*::*LEU2,*	Bailis Lab***
ABX552-8B	*MAT***a**, *ade2-1*, *can1-100*, *his3-11*, *15*, *leu2-3*, *112*, *trp1-1*, *ura3-1*, *rad52*::*TRP1*	Bailis Lab
ABX171-2A	*MAT***a**, *ade2-1*, *can1-100*, *HIS3*, *leu2-3*, *112*, *trp1-1*, *ura3-1*, *rad54*::*LEU2*	Bailis Lab
ABX172-2	*MAT***a**, *ade2-1*, *can1-100*, *HIS3*, *leu2-3*, *112*, *trp1-1*, *ura3-1*, *rad55*::*LEU2*	Bailis Lab
ABX512-37A	*MAT***a**, *ade2-1*, *can1-100*, *HIS3*, *leu2-3*, *112*, *trp1-1*, *ura3-1*, *rad59*::*LEU2*	Bailis Lab
ABX217-3C	*MAT***a**, *ade2-1*, *can1-100*, *leu2-3*, *112*, *trp1-1*, *ura3-1*, *HIS3*, *rad27*::*LEU2*	Bailis Lab
ABX770-38B	*MAT***a**, *ade2-1*, *can1-100*, *HIS3*, *leu2-3*, *112*, *trp1-1*, *URA3*, *mus81*::*TRP1*	Bailis Lab
ABX859-40A	*MAT***a**, *ade2-1*, *can1-100*, *his3-11*, *15*, *leu2-3*, *112*, *trp1-1*, *ura3-1*, *srs2*::*TRP1*	Bailis Lab
ABX828-1C	*MAT***a**, *ade2-1*, *can1-100*, *HIS3*, *leu2-3*, *112*, *trp1-1*, *ura3-1*, *pol3-01*	Bailis Lab
ABX752-3A	*MAT***a**, *ade2-1*, *can1-100*, *his3-11*, *15*, *LEU2*, *trp1-1*, *ura3-1*, *sgs1*::*URA3*	Bailis Lab
TNX73-3B	*MAT***a**, *ade2-1*, *can1-100*, *leu2-3*, *112*, *trp1-1*, *ura3-1*, *his3*::*URA3*::*his3(405bp)*, *apn1*::*TRP1*, *RAD5*	Negritto lab
ABX2125-15C	*MAT***a**, *ade2-1*, *can1-100*, *HIS3*, *leu2-3*, *112*, *trp1-1*, *ura3-1*, *ogg1*::*LEU2*	Bailis Lab
ABX129-3D	*MAT***a**, *ade2-1*, *can1-100*, *HIS3*, *leu2-3*, *112*, *trp1-1*, *ura3-1*, *rad1*::*LEU2*	Bailis Lab
ABX135-2B	*MAT***a**, *ade2-1*, *can1-100*, *HIS3*, *leu2-3*, *112*, *trp1-1*, *ura3-1*, *rad2*::*TRP1*	Bailis Lab
TNX11-9C	*MAT***a**, *ade2-1*, *can1-100*, *leu2-3*, *112*, *trp1-1*, *ura3-1*, *his3*::*ura3*::*LEU2*, *rad4*::*TRP1*, *rad3-G595R*	Negritto lab
TNX74-5B	*MAT*α, *ade2-1*, *can1-100*, *leu2-3*, *112*, *trp1-1*, *ura3-1*, *his3*::*URA3*::*his3 (405bp)*, *rad14*::*KNMX*, *RAD5*	Negritto lab
ABX193-1C	*MAT***a**, *ade2-1*, *can1-100*, *HIS3*, *leu2-3*, *112*, *trp1-1*, *URA3*, *hdf1*::*TRP1*	Bailis Lab
ABX1372-2D	*MAT***a**, *ade2-1*, *can1-100*, *HIS3*, *leu2-3*, *112*, *trp1-1*, *ura3-1*, *rrm3*::*LEU2*	Bailis Lab
ABX773-46B	*MAT***a**, *ade2-1*, *can1-100*, *his3-11*, *15*, *leu2-3*, *112*, *trp1-1*, *ura3-1*, *sae2*::*TRP1*	Bailis Lab

* Rothstein lab, Columbia University.

* Negritto Lab, Pomona College.

** Bailis Lab, Beckman Research Institute of the City of Hope.

### Spot assay of growth fitness

Liquid cultures of *wt* and each mutant strain were grown at 30° with constant agitation in a 96 well plate. Once cultures reached saturation, cells from each sample were serially diluted (1:10–1:100,000) and 3–10 µL of diluted culture was spotted onto agar plates of YES, YPD or SC (synthetic complete) containing 1–2% DMSO with or without a phenol derivative of interest at IC_50-80_. Plates were incubated for 2–3 days at 30°. YES is for fission yeast cell culture while YPD or SC is for budding yeast cell culture.

### Screen of deletion libraries for phenol-sensitivity and resistance

*S. pombe* haploid deletion library of about 2,800 strains (Bioneer, Version 1.0) and the *S. cerevisiae MAT***a** haploid deletion library of about 5000 strains (Open Biosystems) were provided as liquid culture in 96-well plates and stored at -80°. Working copies of these fission yeast or budding yeast genomic deletion libraries were made by inoculating liquid culture of YES or YPD media in replica 96-well plates in the presence of 100 µg/mL or 200 µg/mL G418 and incubating at 30° for several days, according to Bioneer or Open Biosystems manual, respectively. To screen the libraries, cells of each strain from the plates were transferred with a 48-pin replicator and spotted onto duplicate sets of YES or YPD agar plates containing a testing phenol derivative at a chosen IC concentration or solvent alone as a growth control for normalization. We intended to select IC values of each phenol compound at which only about 10% of mutants of the genomic libraries display *wt* growth and more than 10% of the mutants are able to grow (Bimbó *et al.* 2005). The plates were incubated at 30° for 4 days to observe growth differences among the strains. The colony formation pattern of each plate was recorded by a scanner for analysis. The sensitivity of mutants that displayed inhibitory growth in the presence of a phenol derivative was determined primarily based on visual observation.

### Gene ontology analysis of phenol-responsive genes

The gene-sets identified in the genomic screens were analyzed for function enrichment using GoTerm Finder (go.princeton.edu/cgi-bin/GOTermFinder). Only gene ontology (GO) process terms with a corrected p-value < 0.05 were selected. The graphic presentations were constructed by custom scripts to display each GO process as a circle with its size and radial distance to the center proportional to the number of the genes in the group. Each circle is color coded based on its p-value. The significant abundance of specific gene-sets relative to the curated *S. pombe* GO categories (www.pombase.org) (Wood *et al.* 2012) or *S. cerevisiae* (www.yeastgenome.org) was determined by hypergeometric tests using Matlab function hygepdf (k,N,K,n).

### Fluorescence and confocal microscopy

Cells in exponential growth phase were collected and washed 1–3 times with sterile deionized water. *S. pombe* cells were fixed in ice cold 70% ethanol and heat immobilized as described (Forsburg and Rhind 2006). Methods for DAPI (4’, 6-diamidino-2-phenylindole) and calcofluor staining were based on the published protocol (Alfa *et al.* 1993; Forsburg and Rhind 2006) with modifications (Tang *et al.* 2007). To stain cells with both DAPI and calcofluor, 1X DAPI and 1X calcofluor solutions were mixed at a ratio of 7:1, and 5 μL of the mixture was used to stain the fixed cells. An Olympus IX81 motorized inverted fluorescence microscope with magnification of 1000X was used to capture the fission yeast cell images. ImagePro Plus version 6.3 software (MediaCybernetics, Inc., Bethesda, MD, U.S.A) was used for obtaining and processing cell images.

*S. cerevisiae* cells were similarly prepared for fluorescence microscopy and visualized using fluorescent microscope Nikon Eclipse 8Oi. NIS Elements Version 2.3 was used to create Z-stacks at increments of 0.5 µm for visualization of both DAPI and YFP. The images were colored and merged in Adobe Photoshop to look for colocalization of Rad52 foci with the nucleus.

### RNA preparation

*S. cerevisiae wt* cells were grown with or without BHA, BHT, or BPA until the cells reached late log phase (OD_600_ = 1). Cells were harvested and RNA was isolated from 2 mL screw-cap tubes as described (Tang and Edwalds-Gilbert 2016). Cell pellets were resuspended in 0.15 volumes of AE Buffer (50 mM NaAc, 10 mM EDTA, pH 5.5), and then an equal volume of acid-washed glass beads (Sigma) and phenol (Ambion) equilibrated in AE buffer were added. Following vigorous vortex mixing for 10 min, tubes were incubated at 65° for 10 min, interrupted by vortex mixing every 3 min. A volume of chloroform/isoamyl alcohol mix equal to the phenol volume was added and tubes were mixed and centrifuged for 5 min at 9,000 × g. The aqueous layer was transferred to a new screw-cap microcentrifuge tube and 1uL of DNase I was added. The tube was incubated at 37° for 15-30 min and then incubated at 65° for 10 min to deactivate the DNase I. A phenol/chloroform/AE buffer extraction was performed followed by a chloroform wash. The RNA was precipitated by adding 3.0 M NaAc to a final concentration of 0.3 M and 2.5 volumes of ethanol. This solution was mixed and centrifuged or placed in a -20° freezer overnight. The tube was spun for 30 min at 13,000 × g and the RNA pellet was washed using 70% ethanol. The pellet was air-dried and 35 µL of The RNA Storage Solution (Ambion) was added to the pellet. The concentration of the purified RNA was measured using a Nanovue spectrophotometer.

### RT (Reverse transcriptase)-PCR

To synthesize cDNA for RT-PCR, the ProtoScript M-MuLV First Strand cDNA Synthesis Kit (New England Biolabs catalog #: E6300L) was used. First, the RNA/primer/dNTP mix was prepared for each RNA sample by adding 2 µL of total RNA, 2 µL primer DT23VN, 4 µL dNTP mix, and 8 µL nuclease-free water into two separate microcentrifuge tubes for the experiment and the –RT control. The tubes were heated at 70 *°*C for 5 min, spun briefly, and chilled on ice. Then, 2 µL of 10X RT buffer, 0.5 mL of murine RNase inhibitor, 1 µL of M-MuLV reverse transcriptase (except for the –RT control), and 0.5 µL (1.5 µL for the –RT control) of nuclease-free water was added to one tube containing the RNA/primer/dNTP solution for each RNA sample to make up total volume to 20 µL. All tubes containing the cDNA synthesis reaction were incubated at 42° for 1 hr. Then, the tubes were heated at 80° for 5 min. After the incubations, the reaction volumes were brought to 50 µL by adding 30 µL nuclease-free water to each tube.

After cDNA synthesis, *HAC1* mRNA splicing was monitored by PCR and overall yield was measured by actin. The procedure was carried out as previously described (Cox and Walter 1996) with modifications. The HAC1 forward primer used was 5′- AGGAAAAGGAACAGCGAAGG-3′ and the reverse primer used was 5′-GAATTCAAACCTGACTGCGC-3′ (Cox *et al.* 2011); ACT1 forward primer used was 5′-CTGAAAGAGAAATTGTCCGTG-3′ and ACT1 reverse primer was 5′-CTTGTGGTGAACGATAGATGG-3′. The ProtoScript M-MuLV Taq RT-PCR Kit was used and amplification was accomplished in a BioRad icycler. Products were separated on a 1.2% agarose/1X TBE gel containing 2 µL 10 mM Et Br. Controls lacking reverse transcriptase were also included in the PCR amplification reactions. Expression of actin was measured using the same cDNA preparation and indicated primers. The corresponding DNA bands on gel were visualized and captured by a gel demo system under UV light. The band intensity ratios were analyzed by using ImageQuant TL software.

### Data Availability

Supplementary materials include: methods for IC determination (S5) and data digitization of cell growth (S1), raw data of screening genomic deletion libraries (S2-S4, *S. pombe*; S6, *S. cerevisiae*), kinase deletion mutants (S7, *S. pombe*; S8, *S. cerevisiae*), DNA repair mutants (S9), lists of sensitive strains (S10), hypergeometric tests (S11), and GO Terms analysis (S12). Strains are available upon request. Supplemental material available at Figshare: https://doi.org/10.25387/g3.7580054.

## Results And Discussion

To evaluate cellular response to the phenol derivatives BHA, BHT, and BPA, we screened genomic libraries of both fission yeast *S. pombe* and budding yeast *S. cerevisiae*. We identified genes involved in the response, and then explored the cellular processes affected by these compounds to uncover the potential mechanisms required for tolerance of the phenol stress in several aspects: cell growth, morphological variations, genome stability, and cellular detoxification mechanisms such as the ergosterol pathway, V-ATPase pathway and ER-stress response.

### Genomic Screens of S. pombe and S. cerevisiae Deletion Mutants Revealed Important Cellular Processes Involved in Phenol Stress

#### Genes required for cell growth Upon BHA, BHT or BPA exposure:

We determined the dose-dependent growth curves for *wt* cells of both yeasts in liquid cultures exposed to BHA, BHT, or BPA in 1–3% DMSO (S1). From these curves (S5), we calculated the inhibitory concentrations (ICs) of each phenol resulting in 50–80% growth inhibition (Negritto *et al.* 2017). In various types of growth fitness assays, we usually chose a concentration at which about 10% of mutants display *wt* growth and more than 10% of the mutants are able to grow.

**Figure 1:**
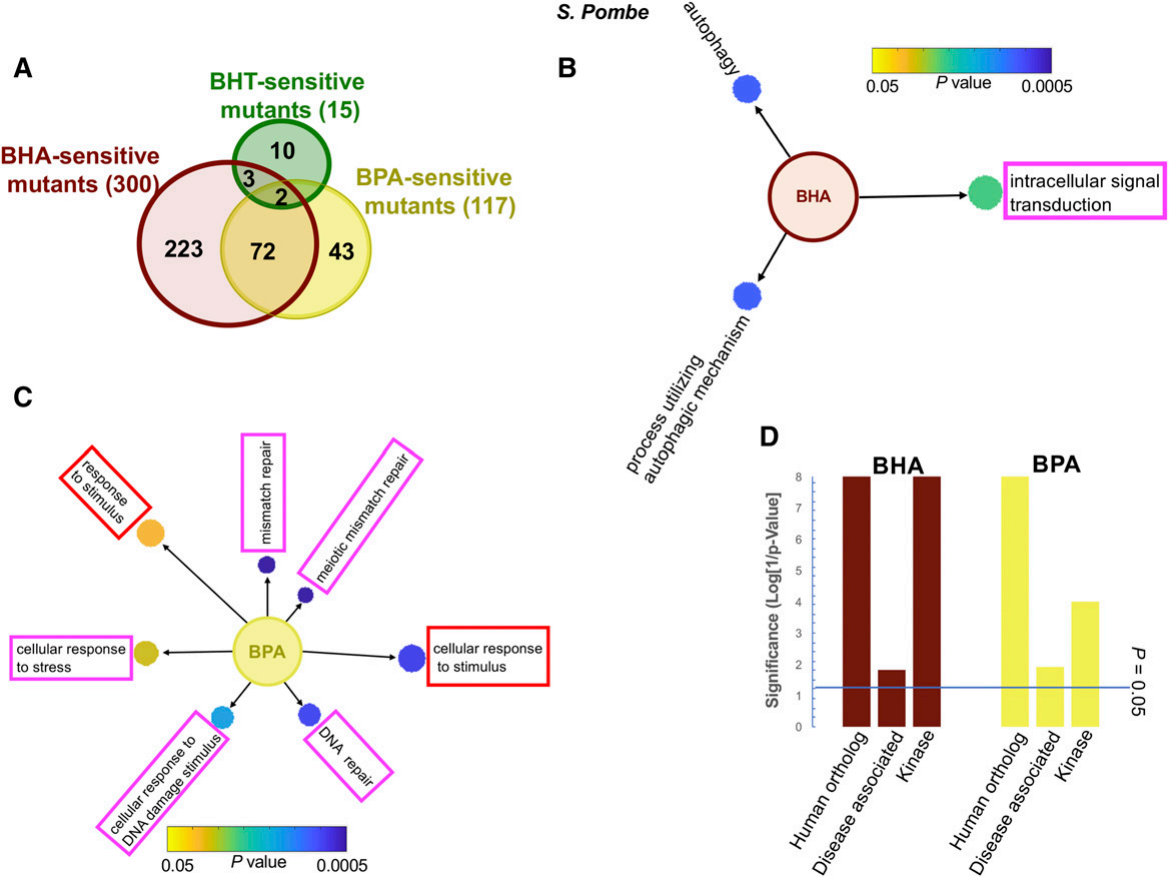

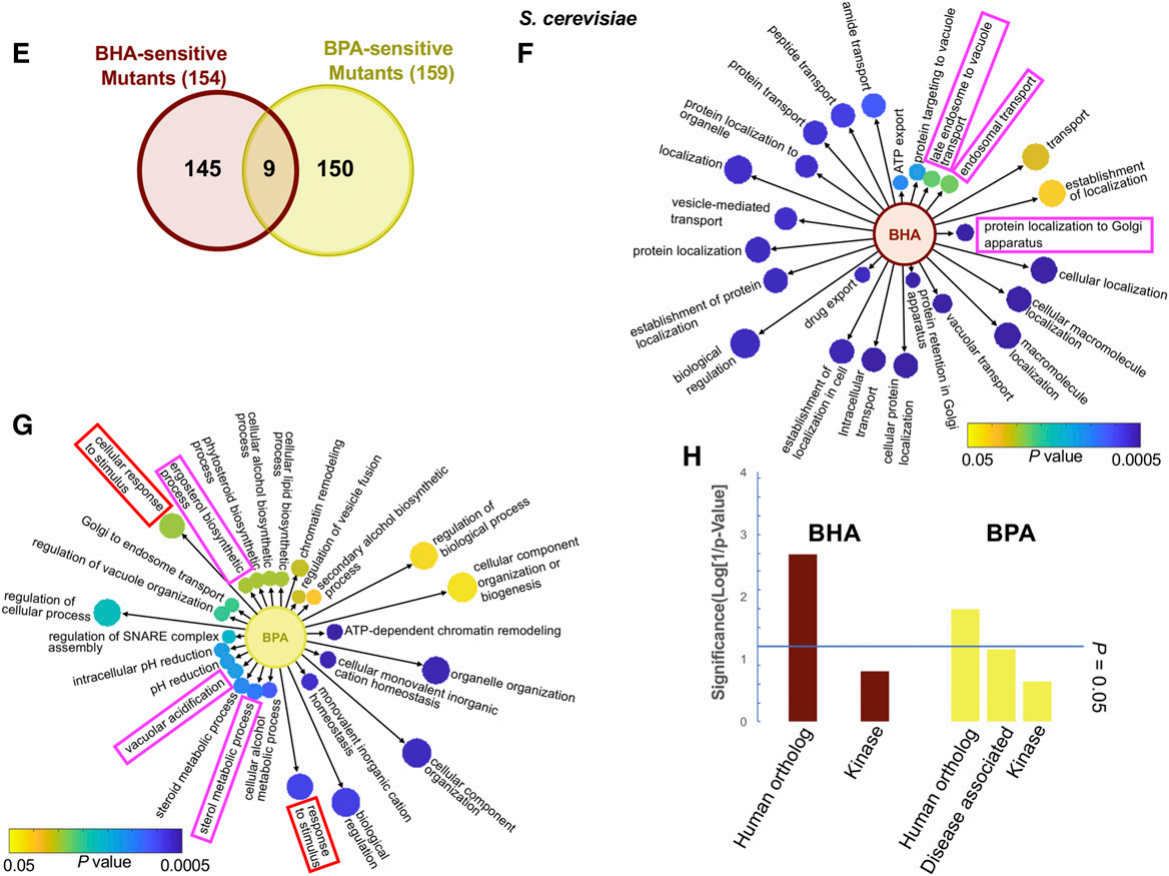
Overview of sensitive mutants to the phenol derivatives from genomic screens of *S. pombe* and *S. cerevisiae*. A. Summary of *S. pombe* mutants sensitive to the phenol derivatives and overlapping sensitivities. B. Main GO terms for biological processes enriched in the dataset of 300 genes involved in the BHA response identified in *S. pombe* genomic screen of deletion mutants. C. Same analysis as in B for the dataset of 117 involved in the BPA response. D. Statistical significance of gene-set enrichment of human homolog, disease-associated, and protein kinase genes in *S. pombe* required for phenol response. E. Summary of *S. cerevisiae* mutants sensitive to the phenol derivatives and overlapping sensitivities. F. Main GO terms for biological processes enriched in the dataset of 154 genes involved in the BHA response identified in *S. cerevisiae* genomic screen of deletion mutants. G. Same analysis as in F for the dataset of 159 genes involved in the BPA response. H. Statistical significance of gene-set enrichment of human homolog, disease-associated, and protein kinase genes in *S. cerevisiae* required for phenol response. In B, C, F, and G, the color and size of each circle represent the p-value and the number of genes in a corresponding term, respectively. Magenta frames indicate pathways that are further analyzed; red frames highlight overlapping processes between the two yeasts. In D and H, the blue line represents p value of 0.05.

For *S. pombe* we carried out at least two trials of the genomic scale fitness test on YES plates containing 1% DMSO with or without BHA (0.3 mM), BHT (0.6 mM), or BPA (0.6 mM) and exhaustively screened about 2,800 *S. pombe* deletion strains (Bioneer *S. pombe* haploid deletion mutant library Version 1.0). The sensitive strain lists were constructed based on visual inspection. Nonviable strains and plate contaminations were eliminated. To verify the results, the colony density was digitized by image processing and the growth score value (GSV) of a deletion strain was calculated as a ratio of colony density of treated over untreated normalized with the *wt* treated over untreated ratio (S1). The GSV’s were cluster-analyzed (Wang *et al.* 2003) at a genomic scale to confirm sensitive and resistant strains (S1-S3). As a result, 300 fission yeast mutants are identified BHA sensitive, 15 strains are BHT sensitive, and 117 strains are BPA sensitive (Figure 1A, S10). Out of the total 353 sensitive mutants identified in the genomic screen of fission yeast deletion mutant library, 63% (223/353) are unique to BHA, 12% to BPA (43/353), and less than 3% (10/353) to BHT (Figure 1A). The genomic screen found more common sensitive genes between BHA and BPA (74) than between BHA and BHT (5). Interestingly, two mutants sensitive to all three phenolic compounds are involved in ergosterol biosynthesis (Figure 1A and S10).

We also screened the *S. cerevisiae MAT***a** haploid deletion library of about 5,000 strains (Open Biosystems) once with 0.5 mM BHA, 0.8 mM BHT, or 1.0 mM BPA to identify sensitive strain modules for further analysis (S6). Growth on plates containing the phenol compounds was compared visually with that on plates containing solvent (DMSO) alone. BHT treatment did not result in any sensitive strains, even when screened with a concentration greater than IC_80_. We identified 154 and 159 deletion strains sensitive to BHA and BPA, respectively (Figure 1E and S10). In *S. cerevisiae* out of the total 304 sensitive mutants, 48% (145/304) are unique to BHA and 49% to BPA (150/304). We sorted the *S. pombe* and *S. cerevisiae* genes deleted in the sensitive strains into functional groups using gene ontology annotations from pombase.org and yeastgenome.org, respectively (S10).

#### Common and distinct cellular processes involved in the response to phenolic compounds:

We analyzed the genes identified in the genomic screens by GoTermFinder (go.princeton.edu/cgi-bin/GOTermFinder) and the data revealed GO Term processes important for the phenol response in both yeasts (S12), unraveling mechanisms for cellular tolerance of the stress. The significant GO Term processes in *S. pombe* include intracellular signal transduction for BHA, cellular response to stress and DNA repair for BPA (Figure 1B and 1C); significant GO Term processes in *S. cerevisiae* include endosomal transport for BHA, ergosterol biosynthesis, and vacuolar acidification for BPA (Figure 1F and 1G). Cellular response to stimulus and response to stimulus are common processes between both yeasts, rich in mismatch repair, double-strand break repair, nucleotide excision repair, and DNA damage checkpoint genes (Figure 1B, 1C, 1F and 1G). Although genes identified in the genomic screens may be distinct between *S. pombe* and *S. cerevisiae*, response pathways are conserved in both yeasts based on GO Term analysis.

Consistently, based on hypergeometric tests (S11), genes involved in the phenol response to BHA and BPA are enriched in human orthologs in both yeasts. The analysis also uncovered significant number of disease-associated genes, as well as the important role of protein kinases in the cellular defense processes in *S. pombe* (Figure 1D).

### Cell Cycle Control and the Phenol Stress-activated Signaling Pathways

#### Protein kinases involved in response to BHA, BHT, or BPA treatment:

**Figure 2:**
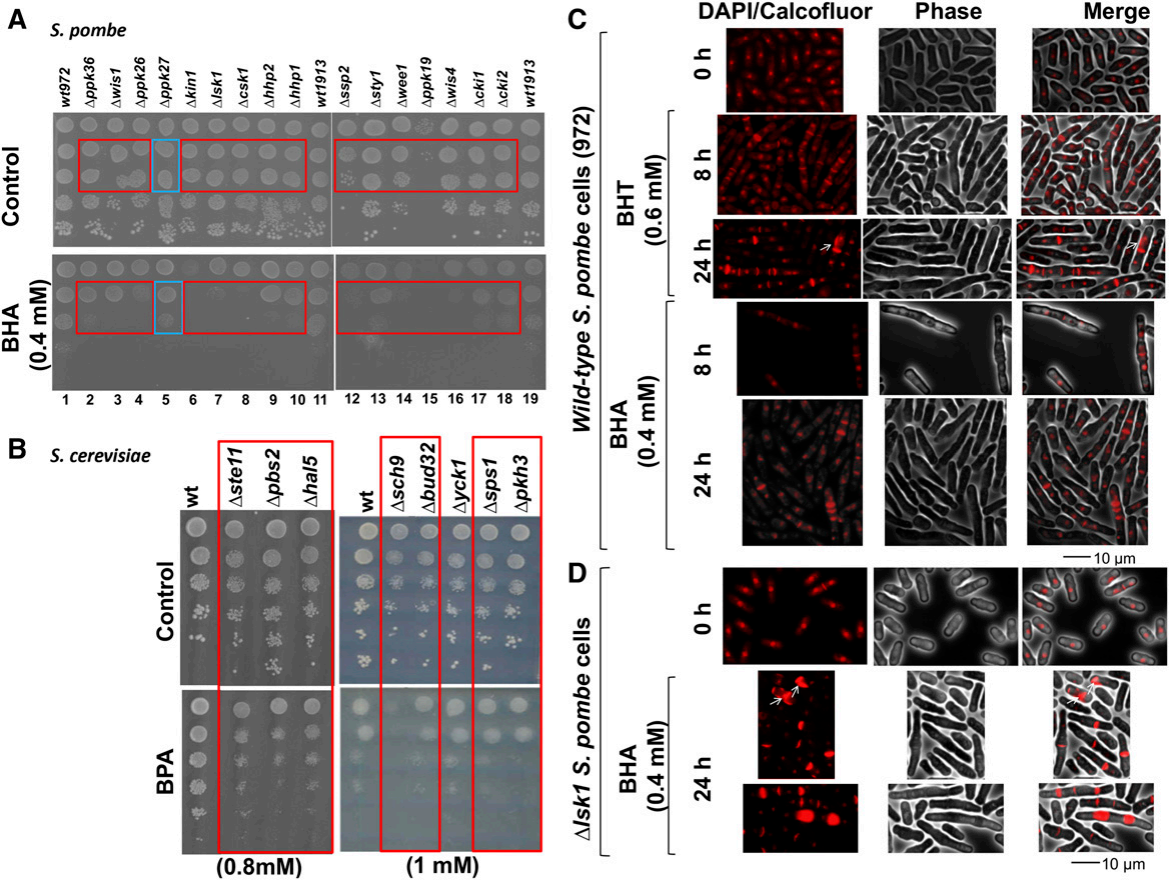
The effects of the phenol derivatives on growth fitness of yeast kinase-deletion mutants and cell cycle control. A. Spot assays confirming the BHA-growth phenotype of the *S. pombe* kinase-deletion mutants. Liquid cultures of each strain were serially diluted with YES to obtain the cell density (cells/ mL) of 1x10^7^, 1x10^6^, 1x10^5^, 1x10^4^, and 1x10^3^. 10 μL of the diluted cultures were spotted onto YES control or 0.4 mM BHA-containing plates. Red frame, sensitive strains; blue frame, resistant strains. *wt*, 972, and 1913. B. An example of spot assay of *S. cerevisiae* kinase-deletion strains exposed to BPA. The 8 kinase-deletion strains were grown overnight in SC media. The cells were diluted to 6x10^6^ cells/mL and then serially diluted by factors of 10 to generate the rest of the spots. 7 µL of cells were spotted onto plates containing SC agar and 1.0 mM BPA or 1% DMSO (control) and allowed to grow for 4 days at 30°. Red frames highlight the sensitive strains. C-D. Morphological changes of *wt S. pombe* cells (972) treated with BHA, or BHT as indicated (C), and *∆lsk1* cells treated with BHA (D). Wild-type 972 or *∆lsk1* were grown in the presence of 0.4 mM BHA or 0.6 mM BHT for 0, 8, or 24 h at 30°. After staining with DAPI and calcofluor, cells were observed under a fluorescence microscope. Arrows point at misplaced septa.

For cells living in a changing environment, rapid proliferation and stress defense represent competing interests for cell survival (Ho and Gasch 2015). Protein phosphorylation by kinases is one of the most notable form of cellular responses to environmental signals and internal processes (Breitkreutz *et al.* 2010; Levy *et al.* 2010). Our genomic screens identified significant number of protein kinases with roles in the cellular response to phenols (Figure 1D). To further investigate this response, we performed growth fitness tests of kinase deletion strains from both *S. pombe* and *S. cerevisiae* in the presence of individual phenol derivatives. One hundred and six protein kinases have been identified in *S. pombe*, of which 17 are essential for viability (Bimbó *et al.* 2005). We examined the growth phenotypes of 85 fission yeast haploid kinase-deletion strains (gifts from Dr. J. Liu, the Genome Institute of Singapore)(Bimbó *et al.* 2005) in response to BHA (0.4 mM or 0.3 mM), BHT (0.6 mM), or BPA (0.6 mM) by pin assays for at least three trials. Strains identified as sensitive were confirmed by serial dilution spot assays. Figure 2A shows an example spot assay of 15 *S. pombe* BHA-sensitive strains and one resistant strain from the collection (Figure 2A). The kinase genes involved are summarized and assigned to functional groups (S10) based on the gene ontology annotations in www.pombase.org. Note that the protein kinases identified have functions in pathways including DNA repair (Hhp1), cell cycle regulation (Lsk1, Csk1, Wee1), and environmental stress-activated signal transduction pathways (Sty1, Wis4, Wis1) (Bimbó *et al.* 2005) (www.pombase.org), indicating that phenolic compounds are detrimental to cells and may cause DNA damage. Moreover, Byr2, which has a role in conjugation and stress response, is required for fission yeast cells to respond to both BPA and BHT stress, while the mitotic cell cycle regulator Wee1, DNA repair protein Hhp1, and vesicle-mediated transport kinase Cki2 are important for cellular tolerance to both BHA and BHT (Figure 6 and S10).

In budding yeast *S. cerevisiae*, we analyzed 110 haploid kinase-deletion strains for response to 1.0 mM BPA, 0.5 mM BHA, and 0.8 mM BHT through four screening trials (S8). Strains exhibiting impaired growth in at least three out of the four trials were deemed sensitive (S10). Figure 2B shows a sample spot dilution assay with seven strains sensitive to BPA (Figure 2B). Similar to the genome-wide screen, no deletion strains showed sensitivity to BHT. Four strains sensitive to both BHA and BPA were identified; one plays a function in meiosis, one in conjugation, and two in vacuolar trafficking (S10).

This study has identified genes encoding protein kinases central to cell cycle regulation in the tolerance of the phenol stress in both *S. pombe* and *S. cerevisiae* (S10). Five kinase orthologs were shared between the two yeasts. For example, missing a protein kinase functioning in conjugation renders *Sc_∆ste11/Sp_∆byr2* sensitive to BPA, while absence of a kinase involved in vacuolar trafficking *Sc_∆vps15/Sp_∆ppk19* results in a strain sensitive to both BPA and BHA. The shared ortholog genes involved in the phenol response between the two yeasts also include *Sc_CTK1*/*Sp_lsk1^+^* and *Sc_BUB1/Sp_bub1^+^*, which encode kinases involved in mitotic control, and *Sc_PBS2/Sp_wis1^+^*, kinases required for stress response (Figure 6 and S10). The genetic screen of the subsets of kinase-deletion strains enhanced the sensitivity of detection and allowed us to uncover common genes between the two yeasts. Finding orthologs provides evidence for a conserved stress response through evolution.

#### BHA and BHT affect cell cycle, cell shape, growth polarity, and cytokinesis:

To study further the physiological effects of the phenolic compounds, *S. pombe wt* cells and BHA-sensitive kinase-deletion strain, *Sp_∆lsk1* (S10) were grown in the presence of BHA or BHT. Cells were collected at various time points and stained with DAPI and calcofluor for imaging by fluorescence microscopy (Figure 2C and 2D). Treatment of *wt S. pombe* cells with both 0.6 mM BHT and 0.4 mM BHA (Figure 2C) led to elongated cells with multiple septa and nuclei, indicating impaired cell cycle progression and cytokinesis. The phenotypes were more pronounced with longer incubation time (Figure 2C, 24 h). The *Sp_lsk1^+^* gene encodes P-TEFb-associated cyclin-dependent protein kinase Lsk1, which phosphorylates RNA polymerase II large subunit Rpb1 (Coudreuse *et al.* 2010). The phenotype of septum distortion in the presence of BHA was exacerbated in the *Sp_∆lsk1* mutant (Figure 2D) compared with that in *w*t cells (Figure 2C). These regulators may be needed for balancing between cell growth and stress defense, as well as for re-entering cell growth states after recovery from stress.

### DNA Repair Genes Constitute Part of the Response Networks to BHA, BHT, and BPA

#### BHA and BPA trigger various components of DNA repair systems:

We identified a number of DNA repair genes in the genomic screening of the deletion libraries of both yeasts (S10). DNA repair process is recognized by GO Term analysis as highly significant for BPA response in *S. pombe* (Figure 1C and S12). More than a dozen genes in DNA damage/repair pathways are required for *S. pombe* cellular response to BHA and/or BPA. Among the BHA- or BPA-sensitive deletion strains identified in *S. pombe* were those with genes known to be important for DNA repair pathways such as base excision repair (BER), nucleotide excision repair (NER), double strand break repair (DSBR), post-replication DNA repair (PRR), non-homologous end joining (NHEJ), and mismatch repair (MMR). We also found genes common to both the BHA and BPA responses (S10). The sensitive gene profiles corresponding to selected phenolic compounds in our studies may allow predictions as to the type of DNA damage caused by a particular phenol derivative.

The response to the phenolic compounds in *S. cerevisiae* followed the same overall pattern as in *S. pombe* (S10): genes in DNA repair pathways including DSBR, NHEJ, NER, MMR, and PRR were identified as involved in cellular tolerance to BHA or BPA as well. Many response genes identified in the two yeasts are members of the same DNA repair pathways, yet, the screening may not be exhaustive; therefore, not all potential genes involved in the response have been identified.

**Figure 3:**
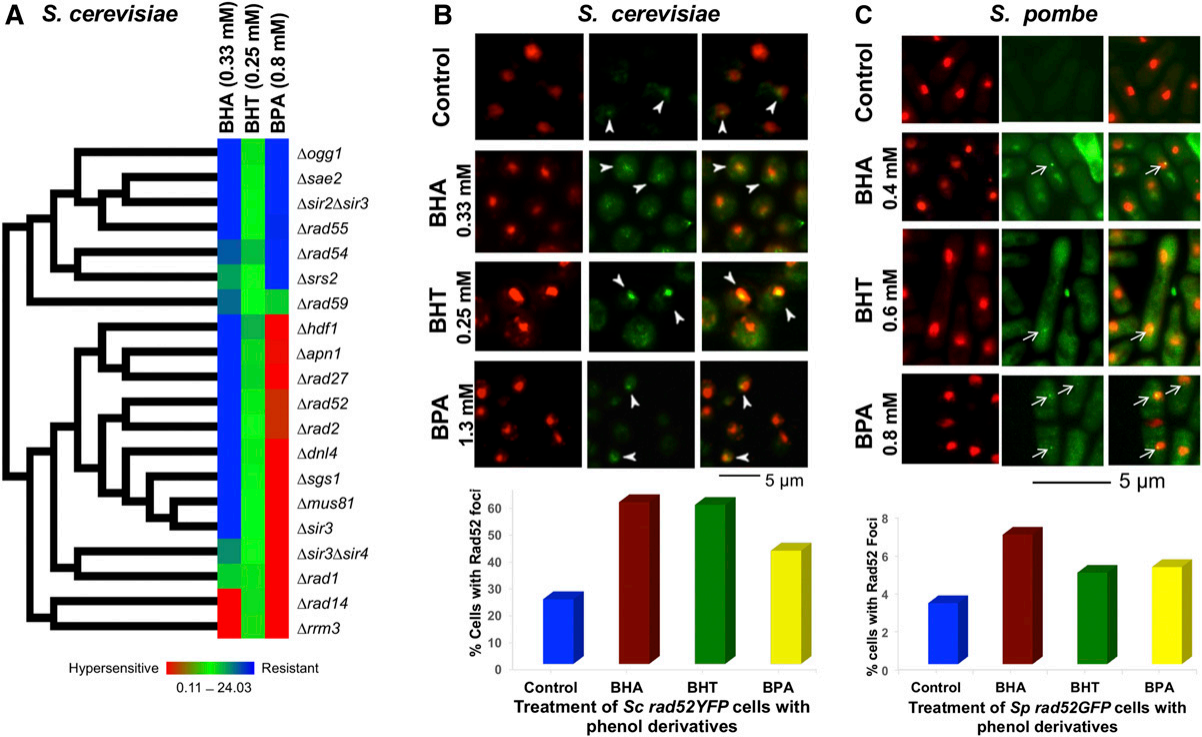
The effects of phenol derivatives on genomic stability. A. Hierarchical clustered growth response of the *S. cerevisiae* DNA repair-deletion mutants to BHA (0.33 mM), BHT (0.25 mM), and BPA (0.8 mM). Growth response profiles of 20 DNA repair-deletion mutants of *S. cerevisiae* to BHA, BHT, or BPA are constructed based on growth fitness assays. DNA repair-deletion mutants of *S. cerevisiae* were grown to saturation in SC media, diluted and spotted onto plates with the same medium containing BHA, BHT, or BPA dissolved in 1% DMSO or 1% DMSO alone (untreated control). The plates were incubated for 3 days at 30°. Each square in the matrix corresponds to a converted value of growth score value (GSV) for each condition. The GSVs of individual strains under different conditions were calculated as ratios of the treated and untreated growth values divided by those of *wt* strains as described in Methods and Materials. The GSVs range from 0.11–24.03, with the values equal or larger than 2.0 being resistant (blue), equal or smaller than 0.5 sensitive (red). Gene/strain names are indicated on the right. Left column is BHA; middle column, BHT; right column, BPA. The growth profiles are clustered based on the GSVs of the corresponding strains. B. The phenol derivatives induce DNA strand breaks in *S. cerevisiae* cells. W3749-14C cells expressing a YFP tagged Rad52 from its genomic locus were grown to exponential phase and treated with 1.3 mM BPA, 0.33 mM BHA, 0.25 mM BHT, or solvent alone (2% DMSO, control) for 4 h at 30°. Cells were then stained with DAPI and visualized under a fluorescence microscope. The arrows point at examples of Rad52 foci. For each treatment, at least 200 cells were counted and the percent of cells with Rad52 foci was plotted. C. The phenol derivatives induce DNA strand breaks in *S. pombe* cells. *S. pombe* cells expressing Rad52 GFP from its genomic locus were grown to exponential phase and treated with 0.8 mM BPA, 0.4 mM BHA, 0.6 mM BHT, or solvent alone (1% DMSO, control) at 30° for 5 h and 10 h. After staining with DAPI cells were observed under a fluorescence microscope. Arrows point at Rad52 foci formed in the cells. A total of 470–709 cells were counted for control and phenol treated samples. The percentages of cells with foci formation were plotted.

Since DNA repair genes are highly enriched in BPA response in *S. pombe* according to GO Term analysis (Figure 1C), we further investigated the genetic response to DNA damage caused by the phenolic compounds. We screened for growth phenotypes a collection of *S. cerevisiae* null mutants for DNA repair genes involved in BER, NER, or DSBR using spot assays (S9). Serial dilutions of a saturated culture of each strain were spotted onto control plates as well as plates containing BHA (0.33 mM), BHT (0.3 mM), or BPA (0.8 mM) and colony growth spots for each strain were quantified by digitization. GSV’s were calculated based on three trials of the spot assays and the average values obtained were used for generating growth profiles of deletion mutants and the corresponding clustering analysis (Figure 3A and S9). In agreement with the genomic screen of *S. cerevisiae* null mutant strains, BHT treatment did not result in visible effects (Figure 3A, middle column). Most DNA repair mutant strains exhibited a growth phenotype to the other two phenols. Out of the 20 null mutant strains, 13 were resistant and two sensitive to BHA (Figure 3A, left column) and 13 were sensitive and six resistant to BPA (Figure 3A right column). The deletion mutant growth profiles look different for each phenolic compound tested, suggesting that the type of damage caused by each phenol is not the same, thereby eliciting a specific repair mechanism. This focused screen with a subset of deletion strains identified more genes involved in the response, confirmed the involvement of the same DNA repair pathways that were identified in the genomic screens, and identified some shared genes in the response to BHA and BPA in *S. cerevisiae* (Figure 3A).

#### BHA, BHT, and BPA increase DNA damage in vivo:

The genomic identification of DNA repair genes in the phenol response prompted us to investigate the effects of BHA, BHT, and BPA on the genomic stability of *S. cerevisiae* and *S. pombe*. Rad52 is one of the key factors in the assembly of the repair machinery around double strand breaks (DSBs), forming the cytological manifestations of repair foci (Misteli and Soutoglou 2009). We therefore used fluorescence microscopy to monitor foci formation in *S. cerevisiae* haploid strain W3749-14C cells expressing YFP-Rad52 fusion protein (gift from Dr. Rodney Rothstein, Columbia University) (Lisby *et al.* 2003) either untreated or treated with BPA, BHA, or BHT for 4 h at 30° (Figure 3B). Rad52 foci were observed in more than 40% of the treated cells compared with only 24% in untreated cells (Figure 3B). Similar experiments were carried out in fission yeast. *S. pombe* cells expressing Rad52-GFP from its genomic locus were treated with BPA, BHA, or BHT at 30° for 5-24 h. Phenol-treated cells display more Rad52 foci than controls (Figure 3C), although foci levels are generally low. The results suggest that phenol compounds may lead to DNA breaks, interfere with DNA replication, or inhibit DNA repair, resulting in increased foci in treated cells. Induction of DSBs is in agreement with results from Negritto *et al.* (Negritto *et al.* 2017), in which cells treated with BHA showed increased levels of homologous recombination leading to genomic deletions.

### Genes in Ergosterol Biosynthesis Participate in Response to BPA

**Figure 4:**
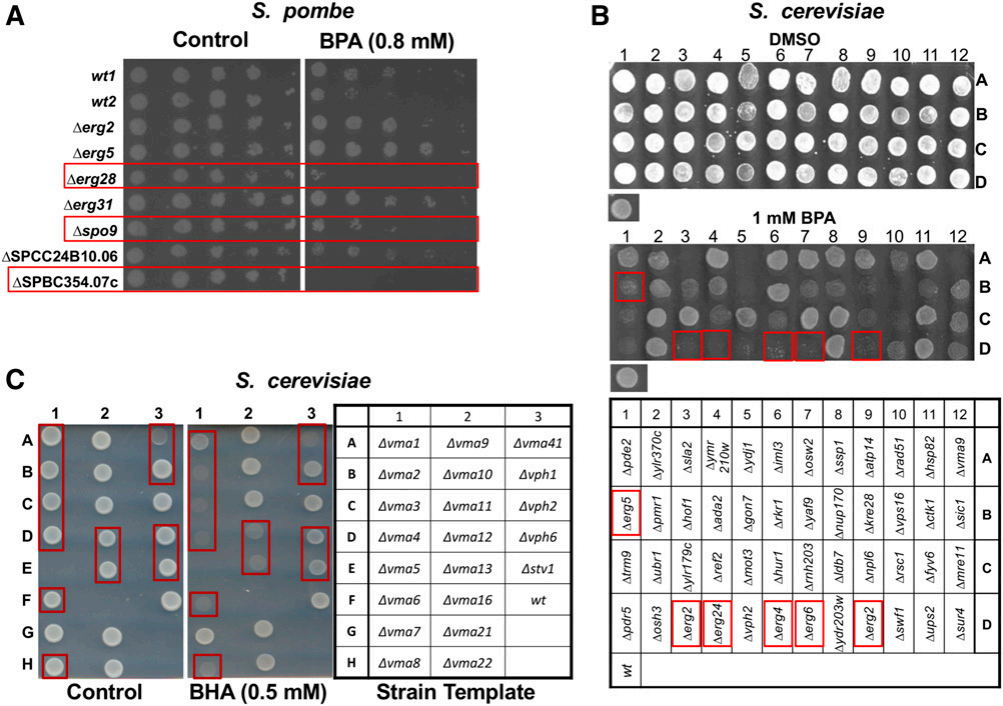
Growth fitness assays of yeast ergosterol- and V-ATPase-related deletion mutants in the presence of the phenol derivatives. A. Fission yeast spot assay of ergosterol-related deletion mutants treated with BPA. *wt* cells and ergosterol-deletion mutants were grown, diluted and spotted onto control plate or treated with 0.8 mM BPA, The plates were incubated for 3 days at 30°. *wt1* is strain *zt501dsk1GFP* and *wt2* is *zt501kic1GFP*. Red frames highlight the BPA-sensitive strains. B. Budding yeast plate growth assays of ergosterol-related deletion mutants treated with BPA. *wt* cells (bottom left spot of each panel) and 44 strains identified as BPA-sensitive in the initial large-scale screen were grown overnight in 96-well plates in YPD and then 6.5 μL of each strain was spotted onto SC plates containing 1% DMSO (top panel) or in addition 1 mM BPA (middle panel). Plates were incubated for 2 days at 30°. The strain layout is indicated (bottom panel). Red frames highlight the sensitive mutants in ergosterol pathway. C. *S. cerevisiae* cells deleted in V-ATPase components are hyper-sensitive to BHA. Twenty-one haploid deletion strains or *wt* were grown in YPD to late log phase (OD_600_ = 1). Cells were then diluted to 1x10^7^ cells/mL. 5 μL were spotted onto SC agar plates containing 0.5 mM BHA (middle panel) or control (left panel) and incubated for 3 days at 30°. Strain layout is indicated (right panel). Red frames indicate strains sensitive to BHA.

The genome-wide screening of both *S. pombe* and *S. cerevisiae* deletion libraries identified genes in the functional group of ergosterol/sterol biosynthesis for the phenol response pathways (S10). GO Term analysis confirmed the significance of the ergosterol synthesis and sterol metabolism in BPA response in *S. cerevisiae* (Figure 1G and S12). To further verify the involvement of ergosterol-related genes in the phenol response, seven null mutants of *S. pombe* each missing a gene involved in ergosterol biosynthesis were analyzed in spot assays in the presence of 0.8 mM BPA in triplicate. Among them three were sensitive to the phenol derivative compared with the *wt* cells (Figure 4A). The proteins expressed from *Sp_erg28^+^* and *Sp_spo9^+^* function in the ergosterol biosynthesis pathway; the gene product of *Sp_SPBC354.07c^+^* plays a role in sterol intermembrane transfer (www.pombase.org). Based on *S. pombe* genomic screen data all three genes when deleted confer sensitivity to both BHA and BHT, while two of them, *Sp_∆erg28* and *Sp_∆SPBC354.07c* display sensitivity to BPA (S10). This spot assay result not only confirmed the genomic data but also added *Sp_∆spo9* to the BPA-sensitive list. Similarly, screening of a subset of budding yeast strains for three times also verified the sensitivity of *Sc_∆erg2*, *Sc_∆erg4*, *Sc_∆erg5*, *Sc_∆erg6*, and *Sc_∆erg24* to 1 mM BPA (Figure 4B). The data further implicate a role of ergosterol biosynthesis in phenol stress.

### Components of the V-ATPase in the Response to BHA

The genomic screening of the two yeast deletion libraries identified genes with functions in vacuolar trafficking (S10). Vacuolar transport and vacuolar acidification are confirmed as significant processes in *S. cerevisiae* for BHA and BPA responses, respectively (Figure 1F and 1G). Vacuolar ATP synthases (V-ATPases) are present in the membranes of vacuoles, endosomes, lysosomes, Golgi-derived vesicles, secretory vesicles, and the plasma membrane of yeast cells (Forgac 2007). V-ATPases are ATP-dependent rotary proton pumps that utilize ATP to move protons from the cytoplasm to the inside of organelles, effectively lowering the internal pH of the organelle (Diakov and Kane 2010). To investigate the observation from the initial genomic screens, we screened a *S. cerevisiae* subset of 21 V-ATPase deletion strains in a plate growth assay in the presence of 0.5 mM BHA in quadruplicate. Results showed that 12 of the 21 strains tested were consistently sensitive to BHA exposure (Figure 4C, red frames). Strains *Sc_∆vma5* and *Sc_∆vma16* did not grow well in either the control or BHA-treated condition (Figure 4C). Most of the sensitive strains are null mutants of genes encoding subunits of V-ATPase; specifically, *Sc_VMA1*, *Sc_VMA2*, *Sc_VMA4*, *Sc_VMA8*, and *Sc_VMA13* encode subunits A, B, E, D, and H of V1 domain of V-ATPase, respectively. Protein products of *Sc_VMA3*, *Sc_VMA6*, *Sc_VPH1*, and *Sc_STV1* are subunits c, d, and two isoforms of subunit a of V0 domain of V-ATPase, respectively.

*S. cerevisiae ∆vma12* and *∆vph6* mutants each lack a protein required for the V-ATPase function (yeastgenome.org), while strain *Sc_∆vma41* (*∆cys4*) is missing cystathionine β-synthase, a regulator of B-ATPase function (Oluwatosin and Kane 1997). These *S. cerevisiae* strains missing a protein in the V-ATPase complex or missing a protein responsible for the function of the V-ATPase complex displayed consistent sensitivity to BHA. Also, screening of kinase-deletion strains confirmed the sensitivity of *∆sch9* (Figure 2B) and *∆vps34* to BPA (S8), which was first identified in the genomic screen (S10). The gene products of *SCH9* and *VPS34* are kinase regulators of V-ATPase assembly and function (Wilms *et al.* 2017). In *S. pombe vph2^+^* encoding a predicted membrane protein of endoplasmic reticulum involved in assembly of the V-ATPase (www.pombase.org) also appears on the lists of genes sensitive to both BHA and BHT (S10), suggesting a conserved role of the V-ATPase in the phenol response.

### BHA and BHT but not BPA Induce UPR (unfolded protein response) in ER

Various changes in physiological conditions and sudden fluctuations in environment, including oxidative stress, can result in ER stress response consisting of UPR, ER surveillance (ERSU), and the ER-associated degradation (ERAD) (Cao and Kaufman 2014). The UPR is an adaptive mechanism that responds to the imbalance between the entry of newly synthesized unfolded proteins and the inherent folding capacity in the ER (Kohno 2010). Consistent with ER’s role in stress response pathways (Babour *et al.* 2010), our genome-wide screen in both yeasts identified deletion mutants of ER-stress-related genes sensitive to BHA and BPA (S10). For example, the Slt2 kinase mediates activation of the ER stress surveillance (ERSU) pathway (Babour *et al.* 2010), and the *S. cerevisiae ∆slt2* strain is sensitive to BHA (S10). Another gene involved in ER stress response, Sc_*IRE1*/*Sp_ire1^+^* (inositol-requiring enzyme 1), encodes a serine/threonine protein kinase acting as a sensor to activate the UPR and is conserved through evolution in eukaryotes (Chen and Brandizzi 2013) (pombase.org). Deletion of the gene renders cells sensitive to BHA in *S. cerevisiae*, and to both BHA and BPA in *S. pombe* (S10).

**Figure 5:**
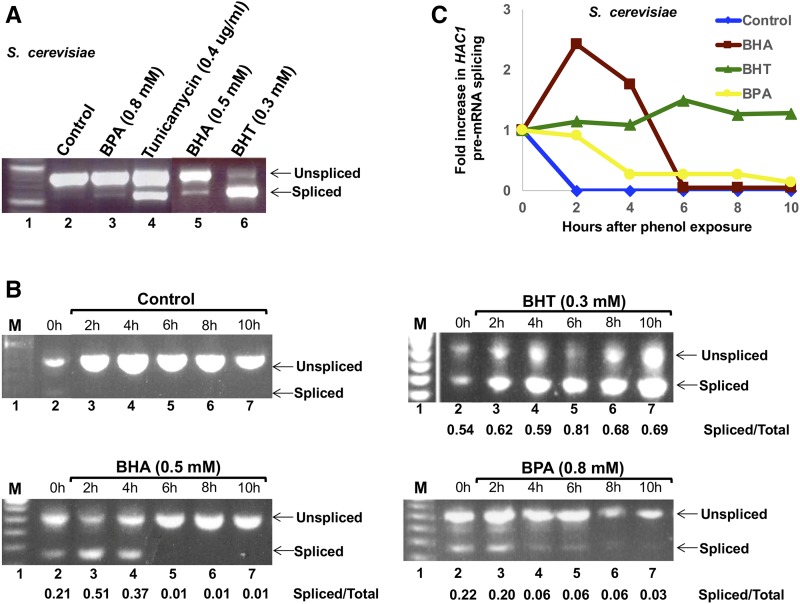
Activation and attenuation of the UPR after cell exposure to phenol derivatives. A. *S. cerevisiae wt* cells were grown in YPD liquid media containing 1% DMSO (control, lane 2), 0.8 mM BPA (lane 3), 0.5 mM BHA (lane 5), or 0.3 mM BHT (lane 6) until the cells reached late log phase (OD_600_ = 1). Cells were also grown in YPD liquid media to late log phase and treated with 0.4 ug/mL Tunicamycin for 1 h (lane 4). After total RNA extraction, *HAC1* mRNA splicing was monitored by RT-PCR. The PCR products were analyzed using electrophoresis with a 1.2% agarose gel. Lane 1, 100 bp ladder. The expected positions of unspliced and spliced pre-*HAC1* mRNA are indicated on the right of the gel. B. Time course of *HAC1* mRNA splicing upon phenol treatment. *S. cerevisiae wt* cells were grown in SC liquid media to log phase and then treated with 1% DMSO (control panel), 0.5 mM BHA (BHA panel), 0.3 mM BHT (BHT panel), or 0.8 mM BPA (BPA panel), for 0 – 10 h, as indicated. After total RNA extraction, *HAC1* mRNA splicing was monitored by RT-PCR. The PCR products were analyzed using electrophoresis with a 1.2% agarose gel. The ratios of spliced *vs.* total *HAC1* transcripts were quantified by ImageQuant. C. Fold increase in *HAC1* mRNA splicing after phenol exposure. The Spliced/Total ratios of *HAC1* mRNA in B were normalized as fold of increase relative to time 0 and plotted *vs.* hours after phenol exposure.

We examined whether the UPR was activated in *S. cerevisiae* cells upon treatment with phenol derivatives by testing splicing of *HAC1* pre-mRNA, which occurs upon ER stress mediated by Ire1 (Babour *et al.* 2010). *wt* cells were grown in liquid media to late log phase containing 1% DMSO solvent, 0.5 mM BHA, 0.3 mM BHT, 0.8 mM BPA, or 0.4 µg/mL tunicamycin as a positive control for UPR induction (Figure 5A). Total RNA was isolated and the splicing of *HAC1* pre-mRNA was analyzed by RT-PCR. Each experiment included a RT control to confirm the lack of genomic DNA in the sample (data not shown). BHA, BHT, and BPA exert distinct effects on UPR in ER as measured by splicing of *HAC1* pre-mRNA in *S. cerevisiae* cells. Cells treated with BHT showed UPR induction similar to the tunicamycin control (Figure 5A, lane 6); BPA and BHA show little UPR activation (lane 3 and 5).

Given the known induction time for the UPR using tunicamycin (Cox and Walter 1996), we performed a time course to monitor the induction of UPR by different phenol derivatives. As expected, no significant splicing occurred over the 10 hr in the presence of the DMSO solvent alone (Figure 5B, control panel). Cells exposed to 0.5 mM BHA showed increased *HAC1* pre-mRNA splicing by the 2-h time point (Figure 5B, BHA panel, lane 3) compared with 0-hour time point (lane 2), indicating the activation of the UPR pathway. After four hours, however, there was clear attenuation of the response (Figure 5B, BHA panel, lanes 5-7), as the spliced *HAC1* RNA transcripts dropped considerably (from 51–37% to 1%). The activation and attenuation of UPR induced by BHA is thus quick and short, represented by a sharp peak of *HAC1* pre-mRNA splicing (Figure 5C). In contrast, BHT treatment resulted in consistent low levels of *HAC1* pre-mRNA splicing starting at 2 h and continuing 10 h after exposure (Figure 5B, BHT panel, compare lanes 3-7 with lane 2). The cells treated with 0.8 mM BPA did not exhibit an increase in *HAC1* pre-mRNA splicing compared with the 0-hour time point (Figure 5B, BPA panel, compare lanes 3-7 with lane 2). Therefore, the three phenolic compounds differ in their capacity of activation and attenuation of UPR in ER as monitored by *HAC1* pre-mRNA splicing (Figure 5B). The results suggest that BHA and BHT may trigger differential levels of UPR in cells, while BPA may not elicit the UPR at all.

## Summary

**Figure 6:**
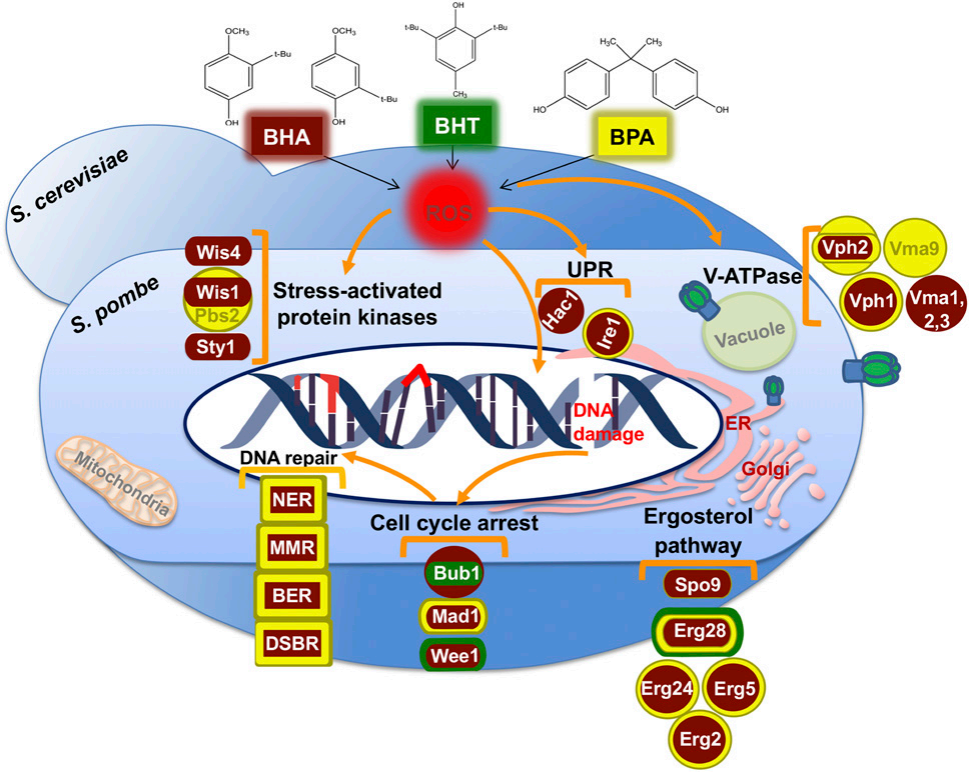
Proposed model of conserved major cellular pathways constituting the networks in response to the phenol stress. Diagrams shown are based on all screens performed in this study presenting main processes and players identified. Dark red, BHA-responsive protein; green, BHT-responsive protein; yellow, BPA-responsive protein. *S. pombe* proteins are depicted as a rod shape and *S. cerevisiae* proteins are depicted as a circle. The phenol derivatives may enter the yeast cells by diffusion or by active transport systems through the membrane. As pro-oxidants, cumulative dosages of these compounds may increase the intracellular ROS, trigger cellular response including stress-activated protein kinase signaling, cell cycle arrest, DNA damage/repair system activation, pH adjustment by V-ATPase, and UPR induction for cell defense. Some proteins in these pathways/processes encoded by genes identified in this study are included as examples. See Results and Discussion for details.

The comparative genomic approach of our studies using divergent *S. pombe* and *S. cerevisiae* offers a global picture of the response pathways to the phenol stress, revealing information about the novel roles of several cellular processes in the cellular defense (Figure 6). First, the genome-scale data presented in this paper offer strong support for the genotoxic action of BHA, BHT, and BPA, potentially leading to genome instability. Second, this study provides the first genetic evidence for targeting major regulators in cell cycle control and cast players linking the Sty1 SAPK (stress-activated protein kinase) signaling pathway to the phase transitions of cell cycle under the phenol stress (Figure 6). Third, components conserved from yeasts to humans and their corresponding functional annotations, allow us to gain an overall perspective of the cellular events taking place upon phenol stress response. These processes include cell cycle arrest to re-allocate cellular energy from cell proliferation to repair processes, DNA repair for error-free inheritance, detoxification through V-ATPase, ergosterol synthesis and UPR for temporarily coping with the stress for ultimate survival (Figure 6). Fourth, hypergeometric tests indicate significant enrichment of genes with human orthologs in both yeasts. In addition, disease-associated genes are enriched in *S. pombe*. The identified major pathways in the two divergent yeasts underscore the cellular processes that are informative for evaluating the human health consequences of phenol derivatives. Finally, the evolutionary spectrum gained by the comparative genomic approach and further examinations in this study enabled us to not only identify the common pathways involved but also recognize potential molecular links in orchestrating the intricate signaling into a concerted response network under the phenol stress.
